# Neopterin, the Cell-Mediated Immune Response Biomarker, in Inflammatory Periodontal Diseases: A Narrative Review of a More than Fifty Years Old Biomarker

**DOI:** 10.3390/biomedicines11051294

**Published:** 2023-04-27

**Authors:** Ondrej Heneberk, Eliska Wurfelova, Vladimira Radochova

**Affiliations:** 1 Department of Dentistry, Faculty of Medicine in Hradec Kralove, Charles University, Šimkova 870, 500 03 Hradec Kralove, Czech Republic; wurfele@lfhk.cuni.cz (E.W.); paulusovav@lfhk.cuni.cz (V.R.); 2 Department of Dentistry, University Hospital Hradec Kralove, Sokolská 581, 500 05 Hradec Kralove, Czech Republic

**Keywords:** neopterin, inflammation, cellular immunity, macrophages, periodontitis

## Abstract

Neopterin is a biomarker of the activation of cellular immunity. The purpose of this review is to summarise neopterin metabolism, methods of its detection, and its role in inflammation, focusing on periodontal inflammatory diseases. This derivative of guanosine is a non-enzymatic product of 7,8-dihydroneopterin oxidation caused by free radicals which protect activated macrophages from oxidative stress. Various methods, usually based on enzyme-linked immunosorbent essay, high-performance liquid chromatography, or radioimmunoassay were developed for the isolation of neopterin. A wide spectrum of diseases and conditions are known to affect neopterin levels, including cardiovascular, bacterial, viral, and degenerative diseases, as well as malignant tumours. Neopterin levels were found to increase in subjects with periodontitis, especially when the oral fluid and gingival crevicular fluid were evaluated. These findings confirm the role of activated macrophages and cellular immunity in periodontal inflammatory diseases. The gingival crevicular fluid and the oral fluid appear to be the most valuable biologic fluids for the evaluation of neopterin levels in periodontitis. For gingival crevicular fluid, neopterin can be determined as the concentration or the so-called total amount. Nonsurgical periodontal treatment was associated with a decrease in neopterin levels, but an increase was also reported, suggesting the possible role of macrophages in the resolution of the periodontal lesion.

## 1. Introduction

Neopterin (Np) is a biomarker of the cell-mediated immune response [1,2]. Np, with the systematic name 2-amino-4-hydroxy-6-(D-erythro-1′,2′,3′-trihydroxypropyl)-pteridine, belongs to the class of pteridines [3]. Np was isolated in 1963 from bee larvae, worker bee [4,5] and royal jelly [5,6]. In 1967, Np was isolated in human urine by Sakurai A. and Goto for the first time [7]. It has been extensively studied in wide-spectrum inflammatory diseases, including viral, bacterial, and parasite infections, cardiovascular diseases, autoimmune diseases, and malignant tumours [8,9,10,11,12,13,14].

This review focuses on the relation between Np levels and periodontitis. The role of Np in immune response is discussed on molecular level as well. The nonspecificity of Np as a cell-mediated immune response is described in a wide variety of diseases and conditions. The articles included in Part 6 were selected by searching “neopterin” and “periodontitis” in the PubMed and Scopus databases. Searching the PubMed database revealed 27 articles. The Scopus database showed 222 positive results between the years 1997–2022. After consideration of inclusion criteria and of the similar results in both databases, 16 articles were included in this review in Part 6. The inclusion criteria were the full texts of the articles available in English that focus on Np levels in different biological fluids in subjects with periodontitis or in other conditions where periodontitis was evaluated by standard periodontal examination/indexes. When the effect of periodontal therapy was evaluated, only studies that evaluated nonsurgical periodontal therapy were included.

## 2. Synthesis of Neopterin

**Figure 1:**
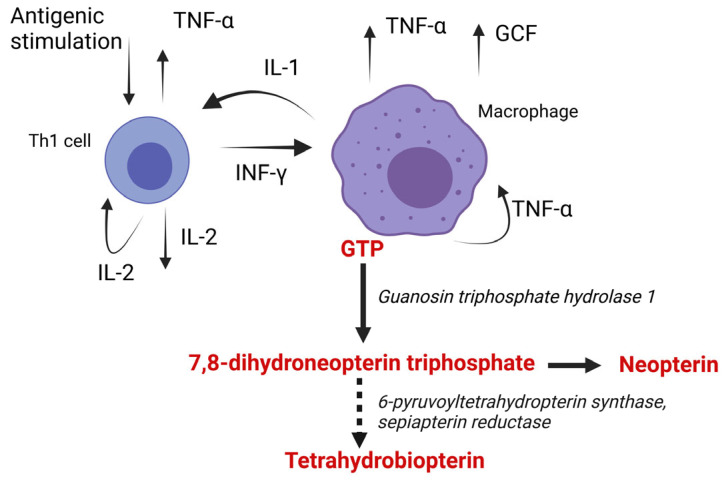
Human macrophages stimulated by interferon gamma convert guanosine triphosphate to 7,8-dihydroneperin. Due to the relative lack of 6-pyruvoyltetrahydropterin synthase, 7,8-dihydroneopterin is not converted to tetrahydrobiopterin but is accumulated in the cells and is non-enzymatically converted to neopterin by free radicals. TNF-α—tumour necrosis factor alfa, IL-1—interleukin 1, IL-2—subtype 1 interleukin 2, Th1 cell—T helper cell, and GCF—colony stimulating factor. Created with Biorender.com.

Np is a metabolite of guanosine triphosphate (GTP) that is transformed to 7,8-dihydroneopterin via cyclohydrolase I. 7,8-dihydroneopterin is subsequently converted to tetrahyrobiopterin by a combined enzymatic reaction with 6-pyruvoyltetrahydropterin synthase followed by sepiapterin reductase [15]. The first enzyme, 6-pyruvoyltetrahydropterin synthase, was found to have low activity in primates, including human´s monocytes/macrophages; 7,8-dihydroneopterin triphosphate is accumulated in these cells [16]. 7,8-dihydroneopterin triphosphate is dephosphorylated by nonspecific phosphatases to 7,8-dihydroneopterin [15], which is converted to neopterin non-enzymatically [17]. 7,8-dihydroneopterin and neopterin are easily transported via cytoplasmatic membrane [17]. A recent study by Janmale [18] showed that 7,8-dihydroneopterin is transported through the equilibrative nucleoside transporter 1 (ENT 1), ENT 2, and concentrative nucleoside transporters (CNT) [18]. In macrophages, cyclohydrolase I activity is upregulated mainly by interferon gamma (IFN-γ) [14]. The main sources of IFN-γ are activated T helper subtype 1 lymphocytes and natural killer cells [14], see Figure 1. Lipopolysaccharides (LPS) and tumour necrosis factor-α (TNF-α) superinduce IFN-γ-mediated neopterin production [19]. Troppmair et al. found that LPS and IFN -α can induce neopterin synthesis independently on IFN-γ [20]. In vivo, concentrations that increased Np concentrations were a hundred times higher for IFN-α than for IFN-γ [20]. 

A study in human umbilical vein endothelial cells revealed that transcription of GTP cyclohydrolase I is driven by activation of the Janus kinase 2/signal transducer and the activator of the transcription proteins (JAK2/STAT) pathway after IFN-γ stimulation and by activation of the nuclear factor kappa B (NF-κB) pathway after TNF-α stimulation [21].

## 3. Biologic Impact of Neopterin

**Figure 2:**
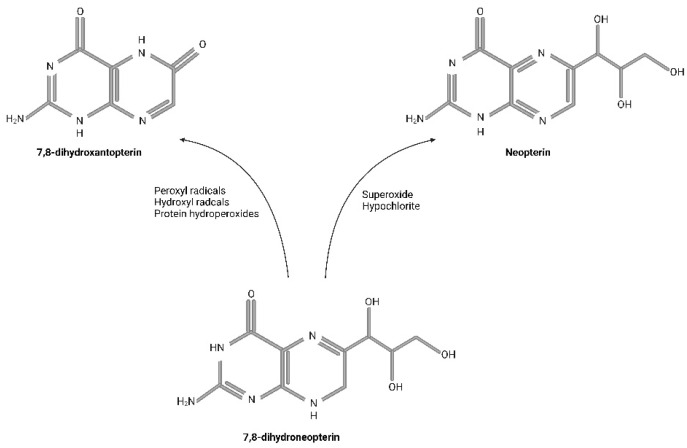
Non-enzymatic products of 7,8-dihydroneopterin oxidation. Superoxide and hypochlorite were found to oxidise 7,8-dihydroneopterin to neopterin. 7,8-dihydroxantopterin is the main product of the reaction of 7,8-dihydroneopterin with peroxyl radicals, hydroxyl radicals, and protein hydroperoxides, while only a small amount of neopterin is formed. Created with Biorender.com.

7,8-dihydroneopterin is a strong reducing agent, free radical scavenger, and chain-breaking antioxidant [22]. It was suggested that activated macrophages produce 7,8-dihydroneopterin to protect themselves at the site of inflammation [22,23]. In cell cultures, 7,8-dihydroneopterin has been shown to scavenge free peroxyl radicals [24], superoxide [25], hypochlorite [26], and hydrogen peroxide [26], see Figure 2. 

7,8-dihydroneopterin was found to be effective in preventing lipid peroxidation and protein hydroperoxide formation [22,24]. Protein hydroperoxides are reactive species that lead to DNA cross-linking and oxidation of cellular thiols and consume key antioxidants such as ascorbates and glutathione [24]. Protein peroxidation can cause enzyme inhibition, affect protein turnover, and lead to accumulation of damaged, poorly degraded proteins [27]. 7,8-dihydroneopterin was found to be capable of reacting and degrading protein hydroperoxides [24]. Although this reaction is slower than the reaction with peroxyl radicals, it may still play an important protective role during respiratory bursts [24]. Lipid peroxidation describes a process of lipid peroxyl radicals and hydroperoxides formation.

Lipids containing double carbon–carbon double bond(s), especially polyunsaturated fatty acids, are attacked by free radicals or non-radical species, and a hydrogen is abstracted from a carbon and oxygen is inserted [28,29]. Extensive lipid peroxidation leads to fragmentation of peroxides and the formation of aldehydes [30]. Even the membrane integrity can be lost due to alteration of its fluidity and membrane-bound proteins can be inactivated [30]. Lipoprotein peroxidation promotes intramolecular or intermolecular protein/DNA cross-linking [30]. These biomolecules can be accumulated within cells with deeply impaired biochemical properties [30]. A study in red blood cell samples showed that 7,8-dihydroneopterin inhibited hypochlorite, hydrogen peroxide, or peroxyl radicals [26].

Np was suggested to inhibit the formation of superoxide mediated by nicotinamide adenine dinucleotide phosphate in rat peritoneal macrophages [25] as a possible feedback loop in inflammation [17].

Antioxidant properties were also studied at the molecular level to prevent the oxidation of low density lipoprotein (LDL) [22,31,32]. LDL particles accumulated in the subendothelial space are oxidised by endothelial cells, smooth muscle cells, and macrophage cells [32]. This process is enhanced in the absence of protective plasma antioxidants, such as tocopherol, ascorbate, urate, apolipoproteins, or serum albumin [33]. The oxidised LDL particles are scavenged and degraded by macrophages [34]. Macrophages accumulate cholesterol esters and foam cells, the hallmark of atherosclerotic lesions, are formed [33,34]. 7,8-dihydroneopterin reduced low-density lipoprotein oxidation induced by Cu^2+^ ions in vitro [22]. The addition of 7,8-dihydroneopterin led to a decrease In both lipid peroxidation and protein hydroperoxides formation [31]. The impact of 7,8-dihydroneopterin on cellular-mediated low-density lipoprotein oxidation was also proved [32]. The addition of increasing concentrations of 7,8-dihydroneopterin caused a progressive decrease in lipid peroxidation in the presence of non-adherent THP-1 cells [32]. In U937 cell culture, oxidative stress induced by peroxyl radicals and 7,8-dihydroneoptein did not prevent lipid peroxidation but was effective in inhibiting protein hydroperoxide formation [24]. Np formation from 7,8-dihydroneoptein was also demonstrated in U937 cell culture and atherosclerotic plaque tissue samples, where it was found to scavenge superoxide [35]. The addition of IFN-γ to adherent macrophage cell culture, such as THP-1 cells, was associated with a significant delay in the rate of LDL oxidation [32]. 7,8-dihydoneopterin also inhibits the expression of the cluster of differentiation 36 (CD 36) via the peroxisome proliferator-activated receptor gamma (PPAR-γ) [36]. CD 36 is the major oxidised low density lipoproteins scavenger receptor, which has a great impact on foam cell formation [36].

Given the potent free radical scavenging of 7,8-dihydroneopterin, it was suggested that the purpose of 7,8-dihydronepterin is to protect activated macrophages against oxidative stress [23] and cell death at the site of inflammation [37]. 

## 4. Neopterin as a Biomarker

Np is considered a biomarker of IFN-γ synthesis [14,38], macrophage activation [39,40,41], and the overall cellular immune system [3,40,42,43]. Np is biologically stable and is not further metabolised in the human body [44,45]. Np is excreted via the kidneys by both glomerular filtration and tubular secretion [39,46].

Generally, pterins have a low brain–blood barrier permeability, so it is unlikely that Np is passively transferred from blood [47]. The estimated serum-to-CSF distribution of Np has been assumed to be as minor as a quotient of 1/40 [48]. In certain conditions, such as Lyme disease or aseptic meningitis, neopterin levels were correlated with cerebrospinal cell count [48,49]. In HIV-associated infections, cellular immune activation was reported to be an important factor associated with brain–blood barrier dysfunction [50].

Neopterin was evaluated in various body fluids, including [10,51], urine, mixed saliva (oral fluid) [10,52,53], gingival crevicular fluid [44,53,54], synovial fluid [55], ascitic fluid [56], cerebrospinal fluid [13], amniotic fluid [57], and pus [58].

Serum neopterin levels were not found to be sex dependent. In most scientific studies, Np concentrations below the cut-off value of 10 nmol/L are considered normal [45]. Several studies confirmed that Np serum levels increase in elderly individuals [59,60,61,62]. In children, increased levels were reported [39], but Plata-Nazar et al. [45] did not find any age dependence between serum neopterin and age, suggesting a cut-off value for normal concentrations lower than 11 nmol/L with a test sensitivity as high as 94.3%. In fetuses, serum Np levels were found to be positively correlated with gestational age, while maternal Np serum concentrations did not correlate [63]. The fetal compartment is isolated from maternal metabolism of unconjugated pterins such as Np [63]. 

In urinary samples, Np is expressed in a ratio with creatinine. These values were found to be higher in women and also decreased in childhood and increased in older individuals [3,45]. A circadian rhythm in urinary NP levels was reported and maximum levels were reached between 7:00 a.m. and 12:00 a.m. [64].

In clinical research, Np levels are usually measured alone. 7,8-dihydroneopterin is not usually assessed in large sample series due to its low stability. The samples must be protected from UV light and stored on ice [17]. In an air-saturated solution at 25 °C, the half-life of 7,8-dihydroneopterin was 60 h [65]. This degradation was accelerated by ultraviolet (UV) light. 7,8-dihydroneopterin is also not fluorescent and its determination requires its oxidation to neopterin with an acidic iodide solution (5.4% I_2_/10.8% KI in 1 M HCl) [66]. Subsequently, the determination of the so-called ‘total Np’, neopterin, and 7,8-dihydroneopterin is performed. This approach requires the measurement of the samples in duplicates. This is problematic in small volumes of samples, such as gingival crevicular fluid, where the median volume in healthy individuals was reported to be 3.86 µL [10] and the sample extraction required an addition of 110 µL of saline [52]. For serum, a HPLC method with mass spectrometry detection that analysed Np and 7,8-dihydroneopterin simultaneously was developed [67]. Given the non-enzymatic Np formation from 7,8-dihydroneopterin, the determination of both Np and total Np gives better information about oxidative stress and immune system activation [17]. Np and total Np were found to be in a constant ratio in serum and urine. In mixed saliva/oral fluid, Np exists mostly in oxidised form [64].

For the determination of Np, various methods were employed. Several high-performance liquid chromatography (HPLC) methods have been developed. HPLC was proposed as a gold standard for Np determination [68]. Commercially available enzyme-linked immunosorbent assay (ELISA) kits are also available. Comparison of HPLC and two ELISA methods revealed the correlation R^2^ = 0.96 for the IBL International ELISA kit (Hamburg, Germany) and R^2^ = 0.48 for the Neopterin ELISA kit (Brahms, Berlin, Germany) compared to the HPLC method [68]. It was recommended not to use trichloroacetic acid for protein precipitation to avoid oxidation of 7,8-dihydoneopterin to neopterin and to instead use acetonitrile [66]. Radioimmunoassay methods were also used to determine Np in serum and urine samples [69], but lower accuracy compared to HPLC was reported in urine samples [39,70]. Polarisation fluoroimmunoessay for neopterin and biopterin determination in urine were also developed [71].

Oral fluid neopterin can be detected by commercially available ELISA kits [44,53,72,73,74]. HPLC methods have also been developed for Np detection alone [75], or in combination with kynurenine, tryptophan, creatinine, and uric acid [52].

Np in gingival crevicular fluid was also detected by commercially available ELISA kits. Only one HPLC method has been developed for the simultaneous analysis of Np in conjunction with creatinine, kynurenine, and tryptophan [54].

## 5. Neopterin in Particular Diseases and Conditions

### 5.1. Neopterin and Cardiovascular System

Neopterin levels were found to be affected by a wide variety of diseases and conditions. In coronary artery diseases and peripheral artery diseases, Np is considered an etiologic and prognostic factor due to the bioavailability of the regulation of nitric oxide (NO) [76,77]. Increased circulating neopterin levels confirm the impact of activated macrophages on vascular rebuild, which, in turn, is able to promote nitro-oxidative stress [78]. Oxidised LDL particles play an important role in the pathogenesis of atherosclerosis and are scavenged in the subintimal space by activated macrophages that are transformed into foam cells [33,34]. The secretion of proinflammatory cytokines such as IL-1 β and TNF-α by activated macrophages plays an important role in the progression and instability of atherosclerotic plaques. This is demonstrated by increased circulating Np levels in the stenoses of both coronary and carotid arteries [78,79,80,81]. Np levels showed a close association with the inflammatory process related to tissue ischemia, illustrated by a lower ankle brachial index [78]. Avanzas et al. [81] presented Np as the strongest predictor of multiple angiographically complex lesions plaque disruption compared to neutrophil count and C-reactive protein levels. Periodontal inflammation was also found to affect peripheral vascular elasticity. Periodontal treatment increased vascular elasticity and was associated with a decrease in circulating Np levels [82]. Parenica et al. [83] also proposed that neopterin together with nitrites, nitrates, and troponin T/I ratio is a predictor of acute kidney injury, a severe and potentially lethal complication of myocardial infraction. Np was found to be independent of all-cause and cardiovascular mortality in subjects who underwent coronary angiography, regardless of angiographic findings and regardless of whether or not individuals presented in a stable or unstable condition (unstable angina, ST-elevation myocardial infarction, non-ST-elevation myocardial infarction) [84].

### 5.2. Neopterin and Bacterial and Viral Diseases

For bacterial diseases, Np was proposed to predict complicated cholecystitis. A 3.34 times higher risk of complications was reported in subjects with Np levels that exceeded the cut-off limit (>14.69 nmol/L) [85]. Np is also a valuable biomarker for the diagnosis of acute appendicitis but was not able to predict the severity of the disease [86]. Np was also proposed as a biomarker for periprosthetic joint infections [55]. Increased Np levels were observed in subjects with tuberculosis and dropped after antituberculosis therapy, suggesting Np as the biomarker of infection activity [87]. IFN-γ mediated immune response plays a principal role in resistance to intracellular pathogens such as Mycobacterium tuberculosis [88]. In addition, other intracellular bacterial infections were associated with elevated levels of Np: Listeria monocytogenes meningitis increased both serum and cerebrospinal fluid Np levels [89] and visceral leishmaniasis affected plasma Np levels [90].

Cellular immunity plays an essential role in viral infection [9]. For example, Np has become well established as a reliable, although unspecific, marker in human immunodeficiency virus 1 (HIV-1) infection, indicating the course and progression of diseases as well as the efficacy of antiretroviral therapy [91]. Np added prognostic information on CD 4 count [92]. HIV-associated neurocognitive impairment was also associated with increased Np levels [93]. High Np levels were also reported to predict worse outcomes in patients with coronavirus disease 2019 [9,94,95] as well as severe acute respiratory syndrome [96]. Increased Np levels were also associated with viral hepatitis A, B, or C [97,98,99]. 

### 5.3. Neopterin and Degenerative Diseases, Autoimmune Diseases, Tumours, and Other Conditions

Np levels in cerebrospinal fluid were affected by neurodegenerative diseases such as multiple sclerosis, neuromyelitis optica, and myelin oligodendrocyte glycoprotein antibody-associated disease and were reported to be useful in differential diagnosis and activity monitoring of these diseases [100]. An association with Parkinson’s disease was also reported [101]. Increasing evidence suggests that chronic low-grade inflammation, associated with higher Np levels, plays an important role in the pathogenesis of neurodevelopmental conditions such as autism spectrum disorder [102]. Increased Np levels were also associated with sarcoidosis [103], systemic lupus erythematodes [104], lichen planus [105] dermatomyositis, and progressive interstitial lung disease [106]. In subjects with rheumatoid arthritis, Np was reported to be a disease parameter but not a marker of disease activity [107,108] in treated patients [107].

Macrophages can play a dual role in cancer control. They represent potent effectors of antitumour immunity. On the other hand, they can contribute to tumour growth and progression [109,110]. Tumour-promoting inflammatory response is currently recognised as an essential feature of cancer [110]. Np was also reported to be a malignant tumour biomarker, such as for colorectal cancer [111], squamous cell carcinoma of the head and neck [11,112,113], melanoma [114], hematologic neoplasms [115], genitourinary tract malignancies [116], and pancreatic cancer [117]. Np was reported to be a risk factor for an increased risk of overall cancer, suggesting a role of IFN-γ–induced inflammation in human carcinogenesis [118].

The ultramarathon race also caused systemic inflammation and higher Np serum levels and was associated with decreased antioxidant capacity [119,120]. The overview of particular diseases and conditions associated with increased Np levels is showed in Table 1.

## 6. Neopterin and Periodontitis

### 6.1. Periodontal Inflammation

Periodontitis is a chronic inflammatory disease of periodontal tissues that leads to their destruction and, when untreated, to tooth loss [121]. Periodontitis is associated with oral biofilm dysbiosis [121]. Gingivitis, the localised inflammation of gingiva, always precedes periodontitis, but not all gingivitis proceeds to periodontitis [122]. Both the oral microbiota and the host inflammatory immune response are essential parts of the pathogenesis of a periodontal lesion. The host immune system, primarily activated to challenge oral microbes, contributes to 80% of tissue destruction [123,124]. 

Dysregulation of the innate and adaptive immune systems can play an important role in the aetiology of periodontal disease [125]. It was hypothesised that a strong innate immune response represented by high IL-12 synthesis is associated with the predominance of the Th1 response, which is characterised by protective cell-mediated immunity and which would manifest as a stable periodontal lesion [122].

**Figure 3:**
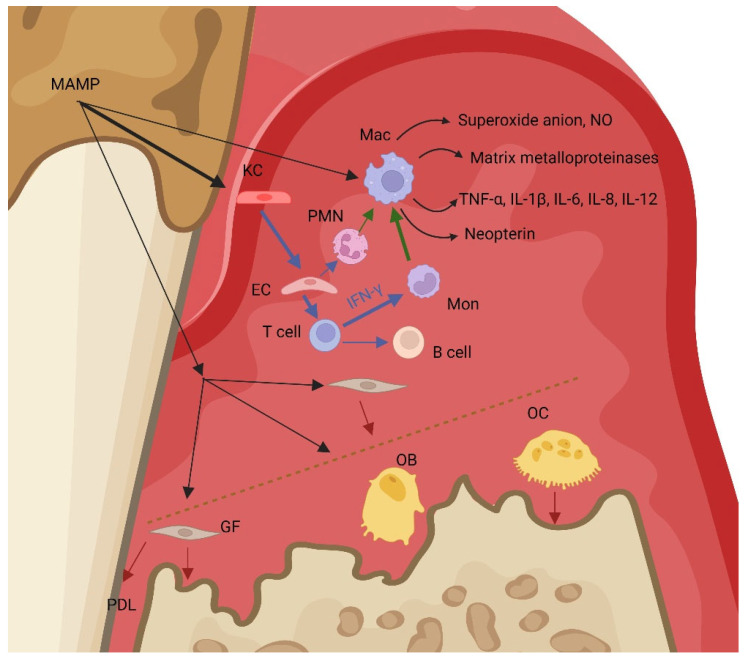
Pathogenesis of periodontitis. In periodontitis, the inflammatory response facing the microbial load leads to destruction of periodontal tissue, including gingival tissue and alveolar bone. The immune response in periodontitis involves both innate and adaptive immunity. Innate cellular immunity (black arrows) represents antigen-presenting cells (gingival keratinocytes), which identify microbes by recognising microbe-associated molecular patterns and activate T helper subtype 1 lymphocytes (blue arrows). Interferon gamma, secreted by T helper subtype 1 and natural killer cells, activates monocytes to polarise to M1 proinflammatory macrophages (green arrows). These cells release metalloproteinases, the hydrolytic enzymes that cleave the different compounds of connective tissue, superoxide anions, and nitric oxide, as well as proinflammatory cytokines TNF-α, IL-1β, IL-6, IL-8, IL-12, and neopterin. Stimulation of osteoclasts leads to bone resorption (red arrows). MAMP—microbial associated molecular patterns, KC—keratinocytes, EC—endothelial cells, PDL—periodontal ligaments, GF—gingival fibroblasts, OB—osteoblasts, OC—osteoclasts. T cell—T helper lymphocyte, B cell—B lymphocyte, Mon—monocyte, Mac—macrophage, PMN—polymorphonuclear cell, NO—nitric oxide, TNF-α—tumour necrosis factor alfa, IL-1β—interleukin 1 beta, IL-6—interleukin 6, IL-8—interleukin 8, IL-12—interleukin 12. Created with Biorender.com.

Macrophages were found to polarise into M1 and M2 phenotypes. M1 macrophages, induced via IFN-γ, are considered proinflammatory immune cells. They secrete a wide variety of hydrolytic enzymes, matrix metalloproteinases, to degrade the different compounds of connective tissue. The M1 macrophages phenotype is associated with the production of matrix metalloproteinase 8, 9 and 12 [44,126,127]. The release of superoxide anions and oxygen and nitrogen radicals also contributes to periodontal tissue damage [128,129]. M1 macrophages are also the source of cytokines, such as TNF-α, IL-1, IL-6, IL-8, and IL-12, which stimulate inflammation [126,127], see Figure 3.

Searching PubMed database and Scopus database revealed 16 studies related to periodontitis impact on Np levels, see Table 2. Neopterin in periodontitis was first studied by Vrecko et al. [72] in urine and oral fluid. Oral fluid neopterin was found to be correlated with the number of affected teeth; no correlation was found for urinary neopterin levels. 

### 6.2. Neopterin in Gingival Crevicular Fluid

For evaluation of the immune response in periodontitis, gingival crevicular fluid (GCF) is essential. This biologic fluid is secreted by the gingival crevice (in healthy periodontal tissues) or the periodontal pocket (in diseased periodontal tissue). Therefore, secretions at the site of inflammation provide the best information on current disease activity. Neopterin GCF concentrations were found to be significantly higher in subjects with periodontitis compared to healthy controls in several studies [130,133,135]. Np GCF concentrations were significantly higher in subjects with periodontitis than in those with gingivitis [130,133]. Furthermore, significantly higher Np concentrations were found in individuals with gingivitis than in periodontally healthy subjects.

For the evaluation of cytokines GCF, the so-called total amount (TA) was also used. TA means the weight or amount of the substance in the whole collected GCF sample. The reason for the evaluation of TA is the increased GCF production in inflamed periodontal tissue caused by increased vascular permeability, which can lead to biomarker dilution. Np TA was proposed to be more valuable to Np concentrations by Ozmeric et al. [44], who found that Np TAs were significantly higher in subjects with aggressive periodontitis compared to the healthy control. The same results were observed by Heneberk et al. [10]. However, the TA assessment has limitations. Deep periodontal pockets accumulate the GCF produced over a longer period. When collected, the volume of the GCF sample represents more resting volume in the deep periodontal pocket than increased GCF production. This was demonstrated by Heneberk et al. [10], who showed that Np GCF TA was higher in subjects with periodontitis than in the healthy control and that Np GCF TA decreased after periodontal therapy. GCF Np concentrations, oral fluid concentrations, serum concentrations, and urine Np to creatinine ratios showed an increasing trend after periodontal treatment. 

After nonsurgical periodontal therapy, both Np GCF concentrations [133,135] and TA [135] were found to drop. Heneberk et al. [10] found GCF Np concentrations to be significantly higher after treatment compared to the healthy control group.

### 6.3. Neopterin in Oral Fluid

Oral fluid is a mixture of saliva, gingival crevicular fluid, and nasopharyngeal secretions [136]. It also contains food remnants [136]. In periodontal research, oral fluid is particularly interesting due to its easy and noninvasive collection [52]. Oral fluid has the potential to replace serum in the evaluation of a wide variety of analytes due to its correlation with serum levels [52]. Compared to GCF, oral fluid provides better information about full mouth periodontal disease activity, while GCF is tooth-specific. Oral fluid Np levels were found to be higher in subjects with periodontitis compared to healthy controls [44,73,131,132]. A significant decrease in oral fluid concentrations have been observed [131], including in a study of premenopausal and postmenopausal women [74]. In a study by Bodur et al. [73], Np was not statistically significant. Heneberk et al. [10] found no significant increase in Np oral fluid levels after periodontal therapy, but after treatment levels were significantly higher than those in healthy controls. 

The source of Np in oral fluid is not clear. Ozmeric et al. [44] stated that the main sources of Np in oral fluid are the salivary glands due to the production of nitric oxide. Heneberk et al. [10] found a fair but significant correlation with serum Np concentrations, suggesting that the serum is the main source. Np OF levels were to be very similar to total Np levels, suggesting that 7,8-dihydroenopterin levels in OF were very low [64]. The small difference in OF and serum Np in total neopterin led to the suggestion that Np in OF originates predominantly in the oral cavity [64].

### 6.4. Serum

No study showed significant differences in serum/plasma Np levels between subjects with periodontitis and healthy controls [10,135]. A significant decrease in Np levels was observed in both pre- and post-menopausal women who completed nonsurgical periodontal treatment [53,134], while Heneberk et al. [10] did not find a significant difference in the levels before periodontal treatment or when compared with healthy controls.

### 6.5. Urine

Only two studies [10,44] found a significantly higher ratio of Np to creatinine in urine. Periodontal treatment was associated with a decrease in the Np to creatinine ratio [53,134], while Bodur et al. [73] found a significant increase. 

### 6.6. Limitation of Neopterin Assessment in Periodontitis

As Np is a very sensitive and nonspecific biomarker of cellular immune system activation, Np reliability may be significantly influenced by concomitant diseases. This was published by Abdel-Haq et al. [105], who found serum Np levels to be significantly higher in subjects with lichen planus compared to a healthy control group. The study group revealed significantly worse periodontal status as described by the community periodontal index of treatment needed (CPITN) [105]. A similar limitation was described by Pink et al. [11], who evaluated oral fluid Np in subjects with oral cancer.

## 7. Conclusions

Most studies concluded that neopterin levels are involved in periodontitis [44,53,72,73,130,133,134,135]. This confirms macrophage activation and its role in the pathogenesis of periodontitis [44,133]. GCF or oral fluid gives the best information about neopterin production in periodontitis. Periodontitis was associated with an increase in neopterin levels and nonsurgical periodontal treatment led to a decrease in neopterin levels, but an increase was also found. This could be explained by increased macrophage activation during the resolution of the periodontal lesion. 

## Figures and Tables

**Table 1:** Particular diseases and conditions associated with increased Np levels.

Article	Disease or Condition	Comment
Zembron-Lacny et al. [77]	Atherosclerosis	Np played a crucial role in the atheromatous process and is useful in monitoring disease severity.
Avanzas et al. [81]	Acute coronary syndrome	Np was a sensitive biomarker for the prediction of the disruption of complex coronary artery lesion.
Nechita et al. [85]	Cholecystitis	Np was found to predict complicated cholecystitis.
Solarino et al. [55]	Periprosthetic joint infections	Np was a promising biomarker for the diagnosis of periprosthetic joint infection.
Saghazared and Rezaei [87]	Tuberculosis	Np levels were found to increase in tuberculosis.
Kaneko et al. [89]	Listeriosis	Listeria monocytogenes meningitis was associated with increased Np levels.
Kip et al. [90]	Visceral leishmaniasis	Np serum levels were found to be higher.
Mildvan et al. [92]	HIV infection	NP predicted disease progression in advanced HIV-1 infection.
Ozger et al. [94]	COVID–19	Np was proposed as an early prognostic biomarker on admission.
Gulcan et al. [94]	Viral hepatitis	Np was proposed as a biomarker for hepatitis-B-related chronic liver disease.
Miyaue et al. [100]	Demyelinating	Np levels in cerebrospinal fluid differed in different demyelinating diseases.
Widner et al. [101]	Parkinson disease	Np levels were increased in advanced Parkinson´s diseases.
Arteaga-Henríquez et al. [102]	Autism spectrum disorder	Increased Np levels were associated with autism spectrum disorder.
Endres et al. [103]	Sarcoidosis	Sarcoidosis was associated with increased Np levels.
Bahrehmand et al. [104]	Systemic lupus erythematosus	Np was proposed to evaluate the progression of systemic lupus erythematosus.
Peng et al. [106]	Dermatomyositis	Increased Np levels were associated with a reduced outcome in subjects with dermatomyositis.
Abdel-Haq et al. [105]	Lichen planus	Increased serum Np levels were reported in subjects with lichen planus.
El-Lebedy et al. [107]	Rheumatoid arthritis	Increased Np levels were found in subjects with rheumatoid arthritis.
Ciocan et al. [111]	Colorectal carcinoma	Np levels in oral fluid were increased in subjects with squamous cell carcinoma in oral cavity.
Pink et al. [11]	Squamous cell carcinoma	Np was proposed as a potential biomarker of colorectal carcinoma.
Weinlich et al. [114]	Melanoma	Np predicted a worse outcome in subjects with melanoma.
Hausen et al. [115]	Haematological malignancies	Urinary Np levels were correlated with tumour stage in subjects with chronic lymphocytic leukemia and with non-Hodgkin’s disease.
Aulitzky et al. [116]	Genitourinary tumours	Higher stages were associated with elevated Np levels in the urine.
Manes et al. [117]	Pancreatic adenocarcinoma	Serum Np levels were higher in subjects with pancreatic carcinoma than in subjects with pancreatitis.
Bizjak et al. [119]	Ultramaraton race	Higher Np levels indicated a decrease in the total antioxidant capacity after the race.

Np—neopterin, HIV—human immunodeficiency virus, COVID–19—coronavirus disease 2019.

**Table 2:** Studies included in the evaluation of the impact of Np on periodontitis.

Study	Key Findings
Abdel-Haq et al. [105]	The purpose of the study was to evaluate Np levels in lichen planus, but serum Np levels were correlated with clinical parameters of periodontitis.
Arjunkumar et al. [130]	GCF Np concentrations were positively associated with periodontal disease.
Bodur et al. [73]	Neopterin levels in oral fluid were found to be increased in subjects with periodontitis before treatment compared to the control group.
Fenol et al. [131]	Oral fluid Np was significantly higher in subjects with periodontitis compared to those in the control group and decreased significantly after nonsurgical periodontal therapy.
Heneberk et al. [10]	Np total amount in GCF and Np to creatinine ratio in urine were higher in subjects with periodontitis. After nonsurgical periodontal therapy, Np in oral fluid increased significantly; in GCF Np concentrations were significantly higher than in the control group.
Mahendra et al. [75]	Np levels in oral fluid were found to be higher in subjects with periodontitis compared to subjects in the control group.
Ozmeric et al. [44]	The total amount of Np in GCF and the concentrations of Np in oral fluid were significantly higher in subjects with aggressive periodontitis compared to the control group.
Patil et al. [132]	Np levels in oral fluid were found to be higher in subjects with periodontitis compared to the control group.
Pink et al. [11]	Comparison of subjects with oral carcinoma with healthy control, but the study group had significantly worse periodontal parameters.
Pradeep et al. [133]	Np concentrations in GCF were significantly higher in subjects with periodontitis compared to the healthy control group.
Prasanna et al. [134]	Np levels in urine and serum decreased significantly after periodontal therapy.
Prasanna et al. [74]	Np levels in oral fluid decreased significantly after nonsurgical periodontal therapy.
Prasanna and Sumadhura [53]	Np concentrations in serum and oral fluid decreased significantly after nonsurgical periodontal therapy.
Ren et al. [82]	Serum Np levels were lower in subjects after nonsurgical periodontal therapy compared to those who received only supragingival calculus removal.
Turgut-Cankaya et al. [135]	Both Np concentrations and total amount were significantly higher in subjects with periodontitis and decreased after nonsurgical periodontal therapy.
Vrecko et al. [72]	Np levels in the oral fluid were significantly higher in subjects with aggressive periodontitis compared to the healthy control group.

Np—neopterin, GCF—gingival crevicular fluid.

## Data Availability

Data sharing not applicable.
